# Microvascular dysfunction in heart transplantation is associated with altered cardiomyocyte mitochondrial structure and unimpaired excitation-contraction coupling

**DOI:** 10.1371/journal.pone.0303540

**Published:** 2024-05-31

**Authors:** Felix Hohendanner, Markus Boegner, Judith Huettemeister, Kun Zhang, Stephan Dreysse, Christoph Knosalla, Volkmar Falk, Felix Schoenrath, Isabell Anna Just, Philipp Stawowy

**Affiliations:** 1 Department of Cardiology, Deutsches Herzzentrum der Charité, Angiology and Intensive Care Medicine, Berlin, Germany; 2 Charité –Universitätsmedizin Berlin, Freie Universität Berlin and Humboldt-Universität zu Berlin, Berlin, Germany; 3 DZHK (German Centre for Cardiovascular Research), Berlin, Germany; 4 Department of Cardiothoracic and Vascular Surgery, Deutsches Herzzentrum der Charité, Berlin, Germany; 5 Department of Health Sciences and Technology, Swiss Federal Institute of Technology (ETH), Institute of Translational Medicine, Translational Cardiovascular Technologies, Zurich, Switzerland; Georgia State University, UNITED STATES

## Abstract

**Introduction:**

Microvascular dysfunction (MVD) is a hallmark feature of chronic graft dysfunction in patients that underwent orthotopic heart transplantation (OHT) and is the main contributor to impaired long-term graft survival. The aim of this study was to determine the effect of MVD on functional and structural properties of cardiomyocytes isolated from ventricular biopsies of OHT patients.

**Methods:**

We included 14 patients post-OHT, who had been transplanted for 8.1 years [5.0; 15.7 years]. Mean age was 49.6 ± 14.3 years; 64% were male. Coronary microvasculature was assessed using guidewire-based coronary flow reserve(CFR)/index of microvascular resistance (IMR) measurements. Ventricular myocardial biopsies were obtained and cardiomyocytes were isolated using enzymatic digestion. Cells were electrically stimulated and subcellular Ca^2+^ signalling as well as mitochondrial density were measured using confocal imaging.

**Results:**

MVD measured by IMR was present in 6 of 14 patients with a mean IMR of 53±10 vs. 12±2 in MVD vs. controls (CTRL), respectively. CFR did not differ between MVD and CTRL. Ca^2+^ transients during excitation-contraction coupling in isolated ventricular cardiomyocytes from a subset of patients showed unaltered amplitudes. In addition, Ca^2+^ release and Ca^2+^ removal were not significantly different between MVD and CTRL. However, mitochondrial density was significantly increased in MVD vs. CTRL (34±1 vs. 29±2%), indicating subcellular changes associated with MVD.

**Conclusion:**

In-vivo ventricular microvascular dysfunction post OHT is associated with preserved excitation-contraction coupling in-vitro, potentially owing to compensatory changes on the mitochondrial level or due to the potentially reversible cause of the disease.

## Introduction

Microvascular dysfunction (MVD) is a hallmark feature of cardiac allograft vasculopathy (CAV) and chronic graft dysfunction in patients after orthotopic heart transplantation (OHT). Microcirculatory resistance has been studied for 20 years in cardiac allografts and is known as a predictor for acute rejections, accelerated CAV and 10-year prognosis [1]. Based on this, coronary flow reserve (CFR) and index of microvascular resistance (IMR) measurements should be performed in routine OHT aftercare [2].

In non-transplanted cohorts, MVD is known to be associated with heart failure (in particular heart failure with preserved ejection fraction; HFpEF), hypertension, coronary artery disease and oxygen supply-demand imbalance during conditions like ischemia with no obstructive coronary arteries (INOCA) [3,4].

Even though only little is known about the cellular mechanisms, the assessment of structural and functional changes on the level of the single cardiomyocytes is often limited due to the nature of the underlying animal model. On a cellular level, impaired mitochondrial respiration has been associated with microvessel disease [5] and treatments targeting mitochondria have shown to confer benefit in experimental ischemia-reperfusion injury [6]. However, our knowledge about the condition-specific underlying mechanisms including effects of microvascular dysfunction and mitochondrial impairment on human cardiomyocyte excitation-contraction coupling is poor [7,8]. Since CAV has a range of ramifications starting with potentially impaired cardiac contractile function, altered myocardial relaxation and increased arrhythmias, a better understanding of the disease and associated conditions appears pivotal. The aim of the current study was to determine the effect of CAV on functional and structural parameters of cardiomyocytes isolated from ventricular biopsies of OHT patients with and without MVD as determined by invasive coronary pressure wire measurements.

## Material and methods

### Study design

Clinical data was derived from a subgroup of a prospective clinical trial (mCAV study) (NCT 05826444) performed at the Deutsches Herzzentrum der Charité from November 1^st^, 2022 to December 31^st^ 2023. The study was approved by the local ethics committee (EA2/133/21) and was conducted in accordance with the ISHLT Ethics statement. Patients provided informed written consent obtained by dedicated study physicians and stored within the clinical research facility on site.

### In-vivo measurements

Patients presented for routine non-invasive and invasive OHT follow-ups at our center. Only individuals (1) free from symptoms of heart failure, (2) with stable electrocardiographic (ECG) and (3) echocardiographic findings and good biventricular graft function without signs of acute or chronic rejection were included in the study. Patients aged younger than 18 or with non-elective presentations for signs of heart failure, palpitations or planned controls after rejection were excluded from the study. Besides clinical investigation, ECG and echocardiography, all patients underwent invasive angiography of the left and right coronary vessels. Moreover, coronary artery microvasculature was assessed using guideline-based coronary flow reserve (CFR) and microvascular resistance (IMR) measurements. In cases with angiograms visually supporting the diagnosis of CAV, resting full-cycle ratio (RFR) and FFR measurements were performed to exclude significant coronary stenosis.

Left ventricular end-diastolic pressure and arterial blood pressure were measured invasively and are available for a subset of patients (see Table 1). Additionally, right ventricular endomyocardial biopsies were obtained (see S1 Table).

**Table 1:** Baseline characteristics.

	MVD n = 8	CTRL n = 6
Age in years	49.9 ± 15.1	49.2 ± 13.1
Sex, male (%)	4 (50)	5 (83.3)
BMI in kg/m^2^	28.1 ± 6.4	26.3 ± 2.9
Years since OHT	10.3 [5.2; 18.5]	7.6 [2.4; 21.5]
LVEDP in mmHg	15 ± 3	17 ± 2
MAP in mmHg	115 ± 3	101 ± 6
SBP in mmHg	141 ±11	128 ± 10
DBP in mmHg	88 ± 3	78 ± 3
History of DS-HLA class II (%)	4 (50)	2 (33.3)
History of quilty	0	2 (33.3)
Everolimus	6 (75)	2 (33.3)
CNI free	0	1
Statins therapy	7 (87.5)	6 (100)

Data is presented as mean ± standard deviation, median [1. Quartile; 3. Quartile] or frequencies (percentage).

Coronary angiography and cardiac biopsies were performed using a transfemoral approach. Target vessels for CFR and IMR measurements using a standard pressure wire (PressureWire X Guidewire; Abbott, Germany) were either the LAD or RCX coronary arteries and CFR/IMR were determined upon administration of heparin and a dose of up to 200 μg Adenosine i.c.. All measurements were performed in accordance with the proposed approach by Santos-Pardo[9] and Toth et al.[10]. In detail, mean transit time (T_mn_) was obtained using cold saline and the distal pressure curves (P_d_) were measured during hyperemia using a PressureWire X Guidewire. IMR was calculated as follows: IMR = T_mn_ x P_d_.

Patients were stratified as either CTRL (IMR <23) or MVD (IMR ≥23) [11]. Cut-offs for CTRL patients were chosen in accordance with Lee and Solberg et al with normal IMRs of 12.4–23.0 and 8.9–22.7, respectively [11,12]. In addition, this cut-off coincides with the median of IMRs in our patient collective (22.5). Upon CFR/IMR assessment, protamine was administered and venous access via the right femoral vein was obtained. Subsequently up to 20 ml contrast agent were administered through a right ventricular pigtail catheter to determine septal positioning of the cardiac biopsy forceps. Right ventricular biopsies were then gathered from the septum using a 7 French sheath (Cordis Corp., Miami Lakes, USA) and a cardiac biopsy forceps (H.+H. Maslanka GmbH, Tuttlingen, Germany). Biopsies were immediately used for in-vitro experiments and standard light microscopy as well as immunohistochemistry were performed to screen for potential cellular or humoral transplant rejection.

### In-vitro experiments

Ventricular septum myocardial biopsies were stored in calcium free buffer solution (including BDM) and single ventricular cardiomyocytes were isolated using enzymatic digestion [8,13,14] in two steps. In a first step, biopsies were incubated in Collagenase XI and Protease XXIV for 10 min at 37°C shaking. In a second step, the biopsy was transferred into a fresh tube containing only Collagenase XI and was incubated for another 10 min before the reaction was stopped with calcium free buffer plus 1% BSA (without BDM). Cells were further dissociated mechanically by pipetting, subsequently centrifuged at 100g and finally placed in 100 μl buffer solution. Calcium was then carefully re-introduced to a concentration of 0.5 mM. In a subset of seven patients, isolated cardiomyocytes from biopsies were viable. Cells from these patients were stained with the fluorescent dye Fluo-4AM using an established protocol and calcium was raised to a final concentration of 3 mM. Upon loading, cells were placed in a small chamber and electrically stimulated (1 Hz) using a pair of platinum electrodes. Subcellular Ca^2+^ signalling during excitation-contraction coupling was measured using confocal microscopy. Confocal line scan images were recorded at 794 lines/s using a 40/1.3 × oil-immersion objective lens (NA = 1.3) with a Zeiss LSM 800 system. The scan line was placed along the longitudinal axis of the cell and cytosolic regions were selected to obtain Ca^2+^ transients. In a subset of cardiomyocytes mitochondrial density was quantified upon staining with Mitotracker/CMTMRos and using a custom-made algorithm and 2D confocal image stacks. Density was calculated from Z-stacks with at least 5 slices at an interval of 1 μm [15,16].

### Chemicals and solutions

Chemicals were obtained from Sigma Aldrich (St. Louis, MO, USA) if not noted otherwise. Fluorescent dyes Fluo-4 and Mitotracker Orange/Red were obtained from Thermo Fisher Scientific (Waltham, MA, USA). Calcium free buffer solution consisted of (in mM): 100 NaCL, 10 KCl, 1.2 KH_2_PO_4_, 5 MgSO_4_, 5 MOPS, 50 Taurin 2 Glucose (for storage and digestion 30 BDM) and was pH adjusted to 7.4 with NaOH.

### Statistical analysis

All data are presented as mean ± standard error mean if not indicated otherwise. Analysis was performed in a blinded fashion. GraphPad Prism was used for statistical inference and plotting (GraphPad Software, San Diego, California USA). To test for group differences non-parametric, unpaired Mann-Whitney tests were used. A p<0.05 indicates significant statistical difference.

## Results

In total, 14 patients were included in the analysis. Mean age was 49.6 ± 14.3 years, mean BMI was 27.9 ± 5.1 kg/m^2^ and most patients were male (64.3%). The median years since OHT was 8.1 years [5.0; 15.7 years] and recorded blood pressures were higher in MVD as compared to CTRL(see Table 1). Mean IMR of the study cohort was 32±7 and mean CFR was 3.9±0.6. Six of 14 patients showed an IMR <23 and served as control.

**Fig 1:**
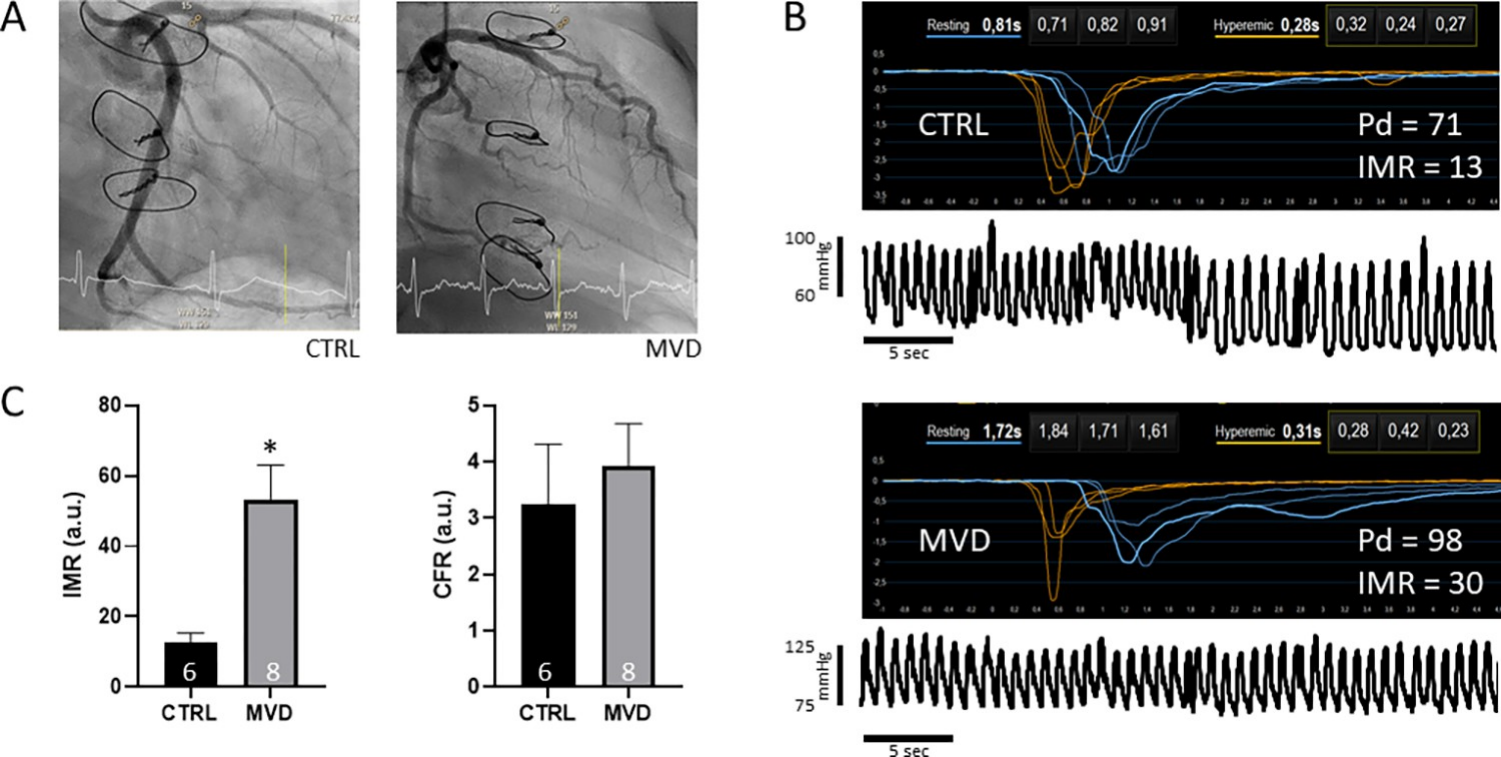
Measuring microvascular dysfunction. A. Left coronary angiogram of a patient without (CTRL) and with (MVD) MVD as determined by coronary pressure wire measurements (IMR; CFR). B. Example measurements of the mean transit time (T_mn_; black background) and the distal pressure curves (P_d_; white background) during hyperemia using a PressureWire X Guidewire. IMR is calculated as T_mn_ x P_d_. C. IMR (left) and CFR (right) in CTRL and MVD patients.

Mean IMR in CTRL and MVD were 12±2 and 53±10, respectively (Fig 1). Time since heart transplantation in patients with MVD was longer as compared to patients without MVD (control group; CTRL). In addition, MVD patients more frequently received everolimus. The blood pressure during the procedure was slightly higher in the MVD group. Two patients showed acute cellular rejection and/or acute humoral rejection upon histological assessment (S1 Table). However, none of the patients showed signs or symptoms of rejection or heart failure and echocardiographic graft function was normal.

In line with that notion, all patients had visually unimpaired coronary angiograms (or normal RFR and FFR) within the coronary pressure wire target vessel and CFR did not differ between MVD and CTRL.

**Fig 2:**
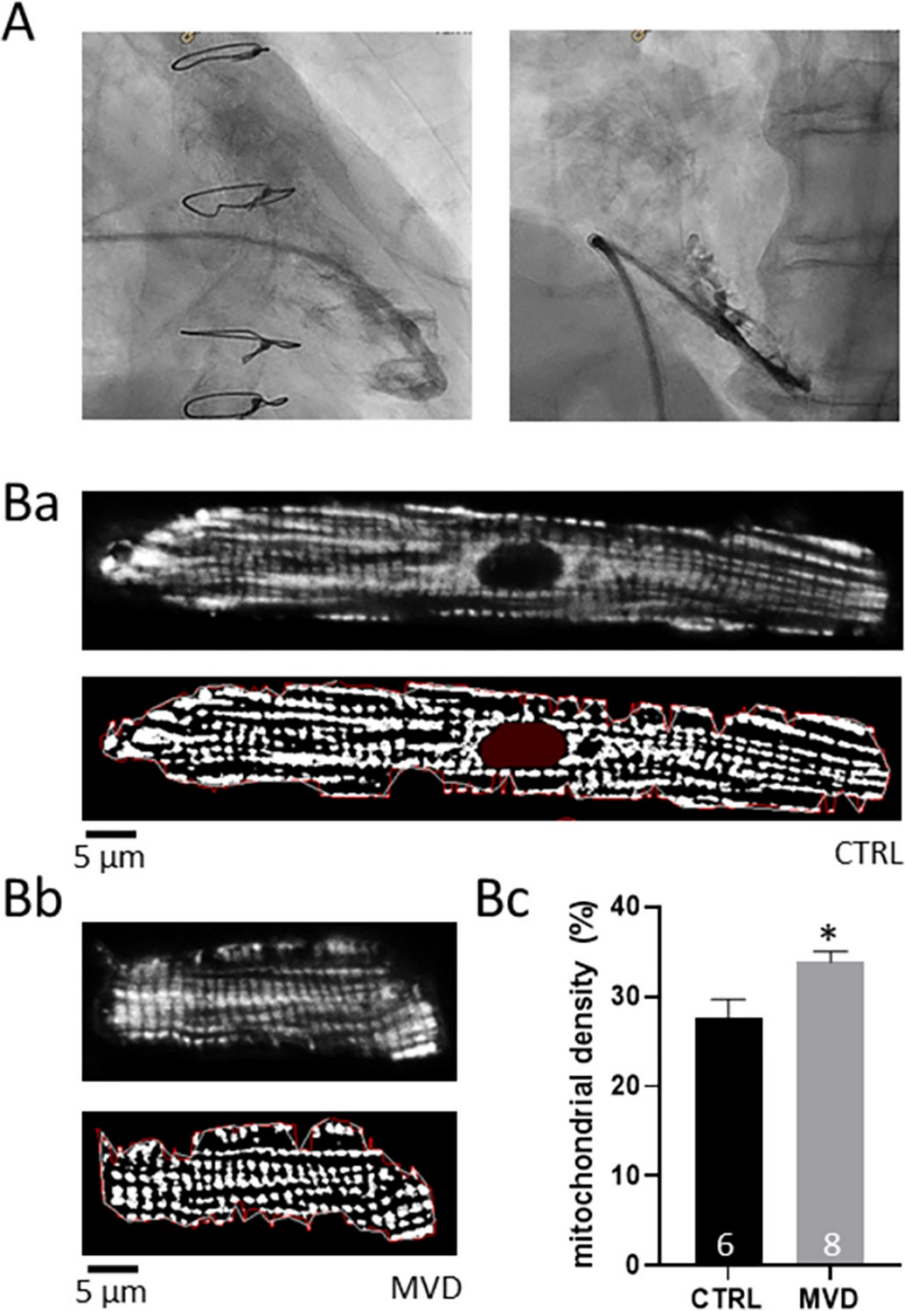
Obtaining RV biopsies and in-vitro quantification of mitochondrial density. A. RV biopsies were obtained upon addition of contrast agent for the positioning of the sheath and subsequent placement of the cardiac biopsy forceps. B. Examples of isolated RV cardiomyocytes (original image: Top; binary image: Bottom) from a CTRL (Ba) and MVD (Bb) patient. Bc. Quantification of the mitochondrial density in CTRL and MVD. *p<0.05.

**Fig 3:**
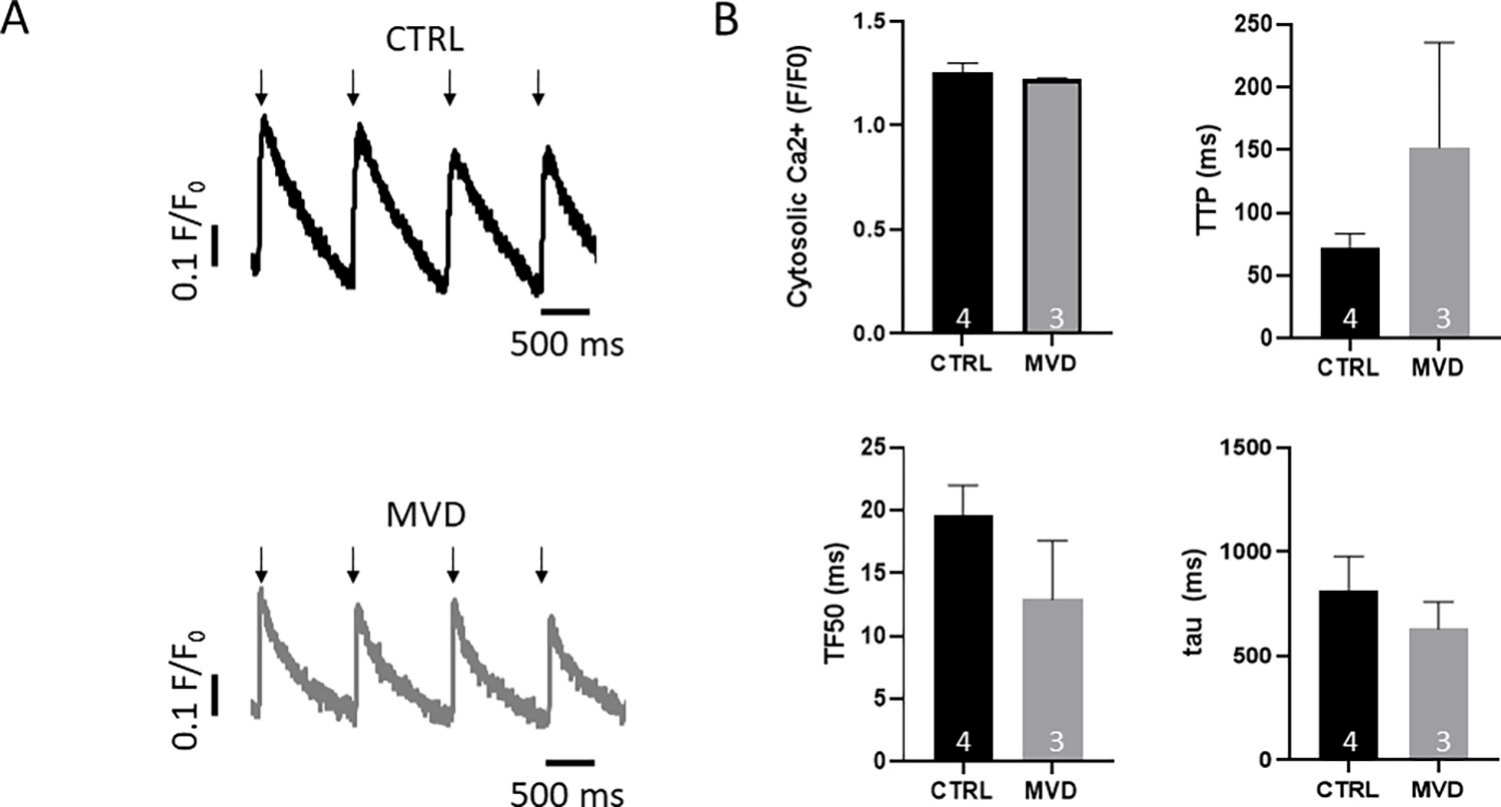
In-vitro Ca^2+^ signaling during excitation contraction coupling in isolated cardiomyocytes. A. Example Ca^2+^ transients during electrical stimulation (1 Hz, arrows) in a CTRL (top) and a MVD (bottom) cell. B. Quantification of the cytosolic Ca transient peak (F/F_0_; top left), time to Ca^2+^ transient peak (TTP; top right), time to 50% of maximal Ca^2+^ release (TF50; bottom left) and the time constant of Ca^2+^ removal (tau; bottom right) in CTRL and MVD patients.

Mitochondria-specific staining and subsequent binary image analysis unveiled an altered mitochondrial density in cells obtained from biopsies of patients with MVD (34±1 vs. 29±2 in CTRL, p<0.05; Fig 2). Mitochondrial density was measured in cells from 6 control and 8 MVD patients. Per patient, a total of 13.8±8.9 and 15.8±9.2 confocal image slices were obtained from 3.2±1.8 and 2.8±1.8 control and MVD cells. To investigate if the altered mitochondrial properties affect cardiomyocyte function, we used cytosolic Ca^2+^ specific dye in a subset of cells. This approach allowed a thorough study of excitation-contraction coupling, including Ca^2+^ release and Ca^2+^ removal, during electrically evoked cellular excitation in isolated ventricular cardiomyocytes from a subset of patients. Ca^2+^ signaling during excitation-contraction coupling was assessed in 3.8±1.3 and 2.3±0.6 cells per patient. With these experiments we detected unaltered Ca^2+^ transient amplitudes (1.2±0.01 vs. 1.3±0.05; a.u. F/F_0_). Ca^2+^ release (i.e. time to peak Ca^2+^) and Ca^2+^ removal (i.e. tau) were also not significantly different between CTRL and MVD, indicating no effect of mitochondrial density on excitation-contraction coupling (Fig 3). Similar results were obtained when excluding patients that showed histopathological signs of acute rejection. These patients had normal and diseased IMRs of 13 and 34 (see S1 Table and S1 Fig).

## Discussion

This study provides novel data on the impact of invasively measured MVD in OHT patients on the corresponding cellular function and structure. We show that in-vitro altered mitochondrial properties and preserved cardiomyocyte excitation-contraction coupling are features of in-vivo MVD in this cohort. Regulation of coronary microcirculation is pivotal to meeting cardiac oxygen demands and MVD is associated with adverse clinical outcomes [17].

Changes observed in MVD produce an environment that might lead to functional or structural adaptation of the cardiomyocyte and neighbouring cells (e.g. fibroblasts [18,19]). Myocardial biomarkers, associated with cellular damage, have been shown to predict adverse outcomes in MVD patients [20,21]. In support of this notion, our results indicate structural changes of the myocardium with particular emphasis on mitochondrial ultrastructure. Interestingly, other groups were able to associate altered mitochondrial fission and permeability transition pore opening with microcirculatory ischemia/reperfusion injury, an acute form of MVD [22]. In fact, mitochondria have long been regarded as potential target organelles for the treatment of ischemia-reperfusion injury [23]. Interestingly, the present study shows for the first time that MVD is associated with unimpaired cardiomyocyte excitation-contraction coupling while mitochondrial density was significantly altered.

In a setting of chronic MVD in non-transplanted patients, impaired oxygen supply, as a hallmark of the disease, might lead to compensatory changes on the level of the single cardiomyocyte and the mitochondria: Evidence for compensatory mechanisms involving mitochondria for the prevention of impaired cardiomyocyte function has been provided by others [24]. In addition, altered mitochondrial structures potentially and profoundly affect Ca^2+^ homeostasis. We and others have shown their direct involvement in a Ca^2+^ related pro-arrhythmogenic environment [25,26]. The fact that Ca^2+^ signalling during excitation-contraction coupling was unimpaired in our patient collective shows the presence of either 1) a compensatory adaptation, 2) an earlier stage of cardiac pathology or 3) due to the different nature of microvascular dysfunction in transplanted patients. Understanding it as a primarily immunological phenomenon, microvascular alterations with consecutive impaired oxygen supply might be a dynamic, reversible process mirroring the current state of alloreactivity rather than a chronic oxygen supply and demand mismatch in other conditions causing MVD.

Indicating the former, there was no clear correlation between IMR and Ca^2+^ transient amplitudes even within the CTRL or MVD collective.

Invasive coronary microcirculation measurements using pressure wires is an established clinical method to derive the function of small arteries, arterioles and is a state-of-the-art procedure in selected patient cohorts [27]. In our patient collective, blood pressure differed between CTRL and MVD, indicating the increased arterial blood pressure might be an underlying pathophysiological driver of the observed MVD. This is in line with the notion that hypertension is a strong trigger of CAV and MVD [28,29]. Despite this, the risk profile between the MVD and CTRL cohorts in our study was comparable. Patients with MVD were more likely to receive everolimus. This is due to our institutional immunosuppressant regime and is explained by the fact that this group had been transplanted for a longer period of time.

In summary, our in-vivo measurements of the cardiac microvasculature in combination with the isolation of single ventricular cardiomyocytes from the very same patients provide novel insights into subcellular mechanisms related to microvessel disease as it can be observed in a variety of clinical conditions (hypertension, heart failure, diabetes, INOCA, transplant vasculopathy).

## Limitations

The small sample size of this proof of principle study as well as the selected subpopulation of heart transplant recipients represents a mayor limitation of the present study. The study might therefore be underpowered to show potential small changes of Ca^2+^ signaling during excitation-contraction coupling. In addition, Adenosine was administered non-systemically, i.e. was administered i.c. (see methods) leading to a higher hemodynamic variability during the IMR measurement.

## Supporting information

S1 Table Endomyocardial biopsies. Patients with humoral or cellular rejection.
(DOCX)

S1 Fig In-vitro data of patients without rejection. Ca^2+^ transient amplitudes (left) and mitochondrial density (right).
(DOCX)

## Abbreviations

BDM 2,3: Butanedione 2-monoxime
CFR: coronary flow reserve
CNI: calcineurin-inhibitor
CAV: cardiac allograft vasculopathy
DBP: diastolic blood pressure
DS-HLA: class II donor-specific human leukocyte antigen class II antibodies
IMR: index of microvascular resistance
INOCA: ischemia with no obstructive coronary arteries
LVEDP: left ventricular end-diastolic pressure
MVD: Microvascular dysfunction
NA: numerical aperture
OHT: orthotopic heart transplantation
SBP: systolic blood pressure
