# Supplementary material for: Microvascular dysfunction in heart transplantation is associated with altered cardiomyocyte mitochondrial structure and unimpaired excitation-contraction coupling
Source: PLoS One. 2024 May 31;19(5):e0303540. doi: 10.1371/journal.pone.0303540 (PMC11142617; doi:10.1371/journal.pone.0303540)
Supplement: S1 Table — Patients with humoral or cellular rejection. (DOCX) [file pone.0303540.s001.docx]

| Table S1: Endomyocardial biopsies | | |
| --- | --- | --- |
| Patient | Acute cellular rejection  (ACR) | Acute humoral rejection  (AMR) |
| 1 | 0 | 0 |
| 2 | 0 | 0 |
| 3 | 0 | 0 |
| 4 | 0 | 0 |
| 5 | 0 | 0 |
| 6 | 0 | 0 |
| 7 | 0 | 0 |
| 8 | 1 | 1 |
| 9 | 0 | 0 |
| 10 | 0 | 0 |
| 11 | 2 | 0 |
| 12 | 0 | 0 |
| 13 | 0 | 0 |
| 14 | 0 | 0 |
| ACR acute cellular rejection; AMR antibody-mediated rejection | | |

Comment: None of the patients showed symptoms or signs of rejection or heart failure. Echocardiographic graft function was normal.
