# Supplementary figures and images for: Microvascular dysfunction in heart transplantation is associated with altered cardiomyocyte mitochondrial structure and unimpaired excitation-contraction coupling
Source: PLoS One. 2024 May 31;19(5):e0303540. doi: 10.1371/journal.pone.0303540 (PMC11142617; doi:10.1371/journal.pone.0303540)

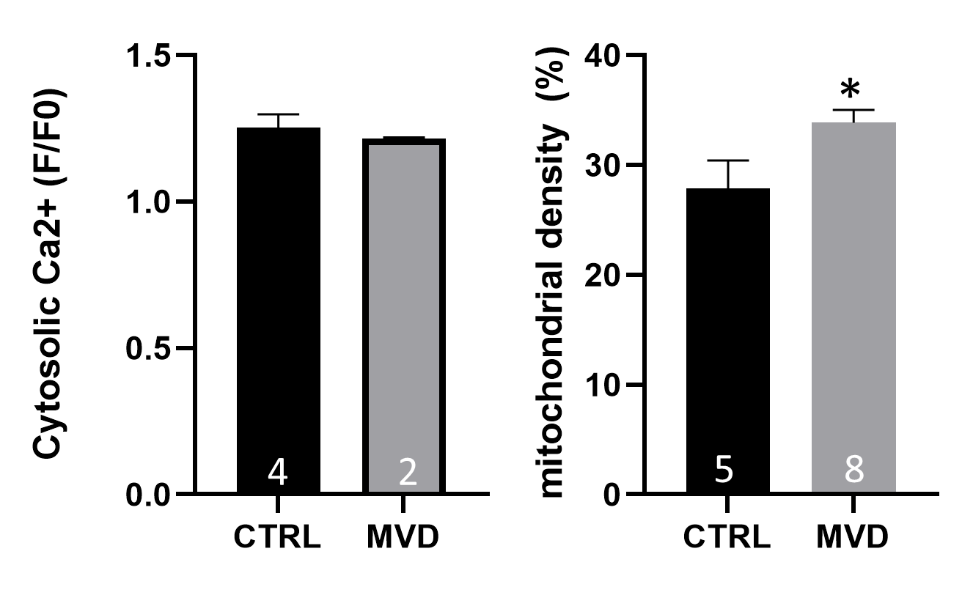

Supplement: S1 Fig — Ca2+ transient amplitudes (left) and mitochondrial density (right). (DOCX) [file pone.0303540.s002.docx]
